# Antitumoral effects of cyclin-dependent kinases inhibitors CR8 and MR4 on chronic myeloid leukemia cell lines

**DOI:** 10.1186/s12929-015-0163-x

**Published:** 2015-07-17

**Authors:** Samuel Troadec, Mélina Blairvacq, Nassima Oumata, Hervé Galons, Laurent Meijer, Christian Berthou

**Affiliations:** Laboratoire de Thérapie Cellulaire et Immunobiologie du Cancer, Université de Bretagne Occidentale, CHRU Morvan, 5 avenue Foch, 29609 Brest Cedex, France; “Protein Phosphorylation and Human Diseases” Group, CNRS, USR3151, Station Biologique, Roscoff, France; ManRos Therapeutics, Hôtel de Recherche, Centre de Perharidy, Roscoff, France; Unité de Technologies Chimiques et Biologiques pour la Santé, Université Paris Descartes UMR-S 1022 Inserm, 4 avenue de l’Observatoire, Paris, France; Current address: Institut Universitaire Technologique, Département de Génie Biologique, Brest, France

**Keywords:** Cyclin-dependent kinases inhibitors, Chronic myeloid leukemia, Cell cycle, Apoptosis, Apoptotic signalling

## Abstract

**Background:**

Although Imatinib mesylate has revolutionized the treatment of chronic myeloid leukemia, some patients develop resistance with progression of leukemia. Alternative or additional targeting of signalling pathways deregulated in Bcr-Abl-driven chronic myeloid leukemia may provide a feasible option for improving clinical response and overcoming resistance.

**Results:**

In this study, we investigate ability of CR8 isomers (R-CR8 and S-CR8) and MR4, three derivatives of the cyclin-dependent kinases (CDKs) inhibitor Roscovitine, to exert anti-leukemic activities against chronic myeloid leukemia *in vitro* and then, we decipher their mechanisms of action. We show that these CDKs inhibitors are potent inducers of growth arrest and apoptosis of both Imatinib-sensitive and –resistant chronic myeloid leukemia cell lines. CR8 and MR4 induce dose-dependent apoptosis through mitochondrial pathway and further caspases 8/10 and 9 activation *via* down-regulation of short-lived survival and anti-apoptotic factors Mcl-1, XIAP and survivin which are strongly implicated in survival of Bcr-Abl transformed cells.

**Conclusions:**

These results suggest that CDK inhibitors may constitute a complementary approach to treat chronic myeloid leukemia.

**Electronic supplementary material:**

The online version of this article (doi:10.1186/s12929-015-0163-x) contains supplementary material, which is available to authorized users.

## Background

Chronic myeloid leukaemia (CML), a myeloproliferative disorder, was the first human disease the onset of which is associated with a specific chromosomal abnormality, the t(9;22)(q34;q11) translocation, known as the Philadelphia chromosome [1]. This translocation creates a novel proto-oncogene giving rise to the constitutively activated protein tyrosine kinase Bcr-Abl responsible for the leukemogenesis of transformed cells and evolution towards blast crisis.

Since a few years, STI571 (Imatinib mesylate; Gleevec), a specific Bcr-Abl tyrosine kinase (TK) inhibitor, has profoundly modified the therapeutic approach of CML and created a big emphasis on development of specific TK inhibitors [2]. However, despite major anti-leukemic effect of STI571 in chronic phase of CML, clinical resistance to STI571 treatment has been observed in patients with advanced phase diseases and has been attributed to mutations in the ATP-binding site of the Bcr-Abl protein (notably T315I mutation) which alter drug binding and thus its inhibitory effects. Moreover, metabolic resistance to STI571 was observed through drug efflux pump Pgp glycoprotein overexpression, Bcr-Abl protein overexpression, BCR-ABL gene duplication [3, 4]. Development of second (Dasatinib, Nilotinib) or third (Ponatinib) generation of Bcr-Abl inhibitors contributed to diminish IC_50_ but not to overcome the major resistance problems, like the T315I mutation [5, 6]. Another cause of disease progression after STI571 treatment is the selection of a Bcr-Abl leukemogenic effect -independent clone that acts through other proliferation signalling pathways such as Gab2 or Cbl/Cbl-b [7, 8]. Therefore, new anti-leukemic strategies need to diversify molecular targets by less selective compounds or by associating alternative selective compounds, in order to prevent escape of resistant malignant clones.

Progression through the mammalian cell cycle is known to be regulated by phosphorylation/dephosphorylation of the retinoblastoma protein (pRb), a process operated by cyclin-dependent kinases (CDKs) which require association with cyclins and phosphorylation to be catalytically active [9]. Based upon this established function of CDKs in cell cycle regulation, and given that approximately 90 % of all neoplasias are associated with CDK hyperactivation [10], several strategies have recently been designed to develop pharmacological compounds that are capable of inhibiting the catalytic CDK subunit, *i.e.* its ATP-binding site. Such chemical CDK inhibitors (CKIs) are extensively evaluated in various diseases, such as cancer chemotherapy, Alzheimer’s disease, or other neurodegenerative disorders, polycystic kidney disease. To date, over 120 CKIs have been identified and characterized (reviewed in [11]) and 10 of which are currently undergoing clinical evaluation as anti-cancer drugs [12]. Purine analogs were among the first low molecular weight inhibitors of CDKs (reviewed in [13]). One of these, (R)-Roscovitine (CYC202, Seliciclib), a potent inhibitor of CDK1, 2, and 5 [14], has reached clinical phase 2 trials against non-small cell lung cancer and breast cancer [15]. Its strong selectivity against a small subset of kinases [16] and limited toxicity and side effects [17, 18] have contributed to its progression through clinical investigations. However, short half-life, strong catabolism and rather weak potencies on CDKs and cell lines (in the sub-micromolar and micromolar ranges, respectively) constitute limiting factors for clinical use. Therefore, second-generation analogues of Roscovitine, conserving initial qualities of the parental molecule, have been developed, guided by the CDK/roscovitine crystal structures to maintain high kinase selectivity and to induce cell death at much lower concentrations [19]. Among which, CR8 (both R- and S- isomers) and MR4 displayed stronger effects on neuroblastoma cells despite rather similar inhibitory activity on CDKs [20, 21]. Based on these previous works, the aim of our study was to evaluate the antitumoral effects of these new CDKs inhibitors in Imatinib-sensitive or -resistant chronic myeloid leukaemia cell lines. Here we report that new Roscovitine-derived CDKs inhibitors R-CR8, S-CR8, and MR4 trigger strong anti-proliferative and cytotoxic effects both in Imatinib-sensitive and Imatinib-resistant cell lines, suggesting that such molecules could join the therapeutic armamentarium against haematological malignancies and chronic myeloid leukemia in particular.

## Methods

### Cell lines

Four human chronic myeloid leukaemia cell lines were used in this study. K562 and KCL22 were kindly provided by Dr Laurence Dubrez-Daloz (University of Bourgogne, Dijon, France), and their Imatinib-resistant respective counterparts K562-R and KCL22-R were furnished by Pr Carlo Gambacorti-Paserini (University of Milan, Italy). Murine pro-B cell line BaF3 transfected with wild-type or T315I P210 Bcr-Abl, used as genetic Imatinib-resistant model, was kindly given by Pr François-Xavier Mahon (Inserm U1035, Bordeaux, France). All cell lines were cultured in RPMI 1640 (Lonza, Levallois-Perret, France) supplemented with 10 % fetal calf serum (FCS) (Lonza), 1 mg/mL L-Glutamine and 100X Penicillin-Streptomycin (Gibco Life Technologies, Saint-Aubin, France). Imatinib-resistant cell lines K562-R and KCL22-R were grown under 1 μM Imatinib-pressure. Twenty-four hours before experiments, these cell lines were washed in PBS and starved from Imatinib.

### Chemistry

Roscovitine was synthesized as previously described [22]. Synthesis of R-CR8, S-CR8, and MR4 was recently described in detail by Oumata and colleagues [19]. Compounds were stored dry and diluted in dimethylsulfoxide (DMSO) as 10 mM stock solutions until use.

### CFSE proliferation assay

Proliferation of the CML cell lines was analysed by flow cytometer using the CFSE staining kit (Invitrogen, Cergy-Pontoise, France). Briefly, cells were stained with 5 μM of CFSE per 10^6^ cells per mL in sterile PBS 1X according to manufacturer’s instructions. One hundred thousands cells were cultured for five days in culture medium and treated with various drugs at indicated concentrations in a final volume of 1 mL. Then, cells were washed, resuspended in 0.5 mL of sterile PBS 1X and ten thousands events were recorded on a Beckman-Coulter XL4 flow cytometer. Control of no proliferation was made treating cells with Actinomycin D (Sigma, Saint-Quentin-Fallavier, France) at 1 μM.

### XTT viability assay

Inhibition of the proliferation of the CML cell lines was confirmed using colorimetric XTT assay kit (Sigma) according to manufacturer’s instructions. Two thousands cells were cultured for one to three days in culture medium and treated with various drugs at the indicated concentrations in a final volume of 200 μL. Then, 20 μL of the XTT formazan dye were added to each sample and plates were reincubated for another 4 h and absorbance was measured at 450 nm on a microplate reader.

### Cell cycle analysis

To assess proliferation inhibition at the cell cycle level, CML cell lines were treated as indicated above for the proliferation assay. At the end of culture, cells were harvested and washed twice in sterile PBS 1X. Five hundred thousand cells were then incubated with 1 mL of DNA staining solution containing 25 μg/mL propidium iodide (PI), 0.1 % sodium citrate, and 0.1 % Triton X-100 (Sigma) for 10 min at 4 °C. PI fluorescence of 20,000 nuclei was analyzed for each sample using a Beckman-Coulter XL4 flow cytometer. The percentage of cells within the G_0_/G_1_, S, and G_2_/M phases of the cell cycle were identified by analysis with the Expo32 ADX™ software (Beckman-Coulter, Villepinte, France).

### Detection of apoptosis

Apoptosis of the CML cell lines was analysed by flow cytometry using the Annexin V-Propidium iodide staining kit (Beckman-Coulter). Briefly, 5.10^5^ cells were cultured for 24 h in culture medium and treated with various drugs at indicated concentrations in a final volume of 1 mL. Then, cells were washed and labelled with Annexin V and propidium iodide according to manufacturer’s instructions. Ten thousands events were recorded on a Beckman-Coulter XL4 flow cytometer. Positive control of apoptosis was made by treating cells with Doxorubicin or Etoposide (Sigma) at 10 μM or 34 μM, respectively.

### Measurement of mitochondrial transmembrane potential

Changes in the mitochondrial membrane potential (ΔΨ_m_) were measured by incorporation of the cationic lipophilic fluorochrome 3,3’-dihexylocarbocyanine iodide (DiOC_6_; Sigma), a cell-permeable marker that specifically accumulates in mitochondria, depending on ΔΨ_m_. CML cells were exposed to the CDK inhibitors for various times and DiOC_6_ at 40 nM was added for the last 30 min at 37 °C in the dark. Then, cells were washed twice with sterile PBS 1X, resuspended in 0.5 mL PBS and analyzed for fluorescence distribution using a Beckman-Coulter XL4 flow cytometer.

### Measurement of reactive oxygen species

Generation of reactive oxygen species (ROS) was measured by increase of fluorescence of the cell-permeable superoxide sensitive probe dihydroethidium (DHE; Sigma). CML cells were stained with 2 μM DHE for 15 min at 37 °C in culture medium without FCS. After washing, 5.10^5^ cells were cultured for 24 h in complete culture medium and treated with CDKs inhibitors for various times or concentrations, as indicated, in a final volume of 1 mL. Then, cells were washed twice with sterile PBS 1X, resuspended in 0.5 mL PBS and analyzed for fluorescence distribution using a Beckman-Coulter XL4 flow cytometer. Positive control of ROS generation was made treating cells with 5 μL of hydrogen peroxyde (Sigma).

### DNA fragmentation analysis

DNA fragmentation was analysed by agarose gel electrophoresis of apoptosis-induced cells’ DNA. Briefly, 2.10^6^ cells were treated or not by drugs at 10 μM or DMSO as vehicle control and incubated for 24 h. Then, DNA was extracted using the QIAamp DNA Mini Kit (Qiagen, Courtaboeuf, France) according to the manufacturer’s instructions. Twenty μL of DNA extracts were ran on 1 % agarose gel at 5 V/cm for 3 h and observed under UV light. Image acquisition was done using the BioXCapt software (Vilber-Loumat, Marne-la-Vallée, France).

### Caspase 3 cleavage detection

Caspase 3 cleavage in treated cell lines was detected by flow cytometry using a specific Alexa Fluor 488-anti-caspase 3 cleaved fragment antibody purchased from Cell Signaling Technology (Ozyme, Saint Quentin en Yvelines). Briefly, 5.10^5^ cells were cultured for 24 h in culture medium and treated with various drugs at indicated concentrations in a final volume of 1 mL. Then, cells were washed and labelled with antibody according to the manufacturer’s instructions. Ten thousands events were recorded on a Beckman-Coulter XL4 flow cytometer.

### Preparation of cell lysates

For each cell line, 30.10^6^ cells were washed in sterile cold phosphate-buffered saline 1X, centrifuged for 5 min. at 1000 g. Cells were then resuspended in culture medium and treated with Imatinib, Roscovitine, R-CR8, S-CR8 or MR4 at 10^−5^ M for up to 24 h. A non-treated control was also done for each time. When time was reached, cells were centrifuged for 5 min. at 1000 g, resuspended in cold sterile PBS 1X, and transferred to 1.5 mL tubes. Tubes were centrifuged, supernatants were discarded and pellets were resuspended in 300 μL of Lysis Buffer (Tris 50 mM, NaCl 140 mM, EDTA 1 mM, Na_3_VO_4_ 1 mM, Triton X-100 1 % v/v, proteases cocktail inhibitor 2 % v/v, pH 7,5) and incubated on ice for 30 min. Tubes were then centrifuged for 10 min at 14,000 g and supernatants were collected as cell lysates in well-identified tubes, quantified for protein amount (Uptima BC Assay Protein Quantitation, Interchim, Montluçon, France) and stored at −80 °C until analysis.

### Subcellular fractionation

Cytosolic and mitochondrial fractions were prepared from cells treated as above using the Mitochondria Isolation kit for cultured cells from Pierce Biotechnology (Brebières, France), according to the manufacturer’s instructions.

Nuclear fractions were obtained using a homemade nuclear lysis buffer containing Hepes 10 mM, NaCl 500 mM, Triton X-100 1 %, glycerol 10 %, NaVO4 1 mM, PMSF 1 mM, RNase 2 % and proteases inhibitors cocktail (pepstatin, aprotinin and leupeptin) 1 μg/mL, pH 7.5. Lysis buffer was applied on pellets resulting from the first centrifugation of subcellular fractionation with the Mitochondria Isolation kit. Samples were sonicated 5–8 times for 30 s each with 1 min pause, then centrifuged at 14,000 rpm for 15 min at 4 °C. Supernatants were saved as nuclear fractions and pellets were discarded.

### Treatment with caspases inhibitors

When caspases inhibition was required, CML cell lines were first pre-incubated with desired caspase inhibitors (R&D Systems, Lille, France) or vehicle (DMSO) for 1 h, and then exposed to treatment with the tested CDK inhibitors as described for each experiment.

### Western-blotting

Equal amounts of proteins (20–30 μg) were resolved using SDS-PAGE (Bio-Rad Laboratories, Marnes la Coquette, France) and electrotransferred onto PVDF membranes. The membranes were blocked with 5 % semi-skimmed milk in PBS-Tween 20 (0.1 %) at room temperature for 1 h, washed three times in PBS-Tween 20 for 10 min each, and probed with the appropriate dilution of primary antibody in 1 % semi-skimmed milk in PBS-Tween 20 overnight at 4 °C. The membranes were washed three times with PBS-Tween 20 for 10 min each, and then incubated with a 1:1000 dilution of HRP-conjugated secondary antibody in 1 % semi-skimmed milk in PBS-Tween 20 at room temperature for 1 h. The membranes were finally washed three times in PBS-Tween 20 for 10 min each before revelation. All antibodies used were purchased from Cell Signaling Technology except for PSTAIR (Chemicon Merck-Millipore, Guyancourt, France), PU.1 (Santa Cruz Biotechnology, Heidelberg, Germany) and actin (Sigma), and were employed according to manufacturers’ instructions. HRP-conjugated secondary antibodies were obtained from Sigma or Cell Signaling Technology and used to detect protein labelling by the Amersham ECL Plus Western Blotting Detection Reagents kit (GE Healthcare, Saclay, France). Results were acquired by a Vilber-Loumat camera and analysed using the Chemi-capt software (Vilber-Loumat).

### Statistical analyses

Results were expressed as means ± standard deviation of three independent experiments. Statistical analyses were performed using the paired two-tailed Student’s *t*-test. Statistical significance was accepted at *p* < 0.05.

## Results

### CR8 and MR4 exert more potent antiproliferative effects than R-Roscovitine in CML cell lines

**Fig. 1:**
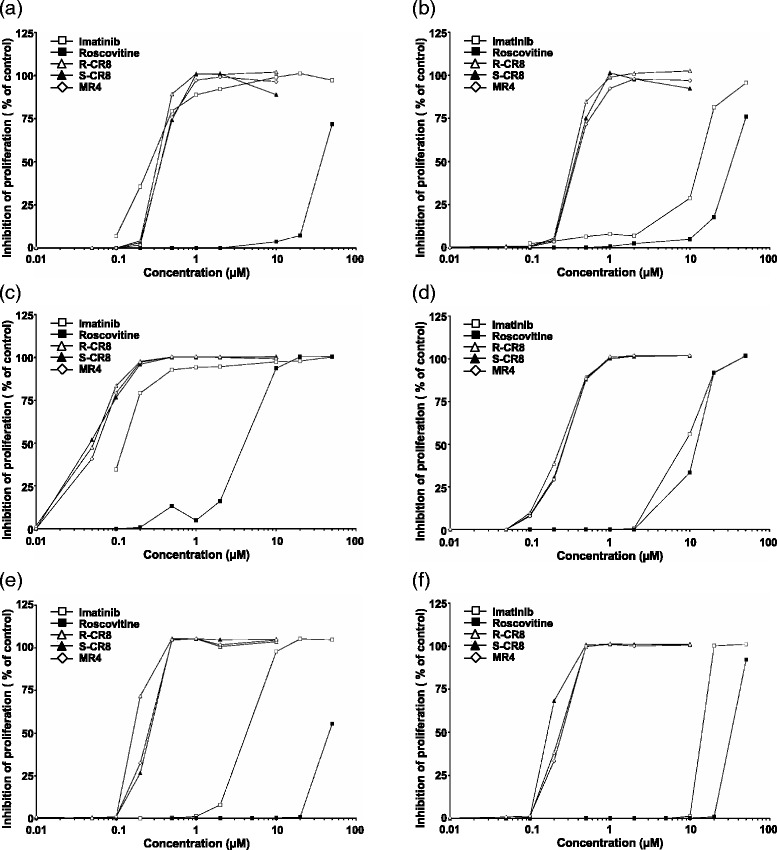
Inhibition of proliferation of Imatinib-sensitive and –resistant CML cell lines. Imatinib-sensitive K562 (**a**), KCL22 (**c**), and BaF3 Bcr-Abl WT (**e**) and Imatinib-resistant K562-R (**b**), KCL22-R (**d**), and BaF3 Bcr-Abl T315I (**f**) CML cell lines were CFSE-labelled and treated with increasing concentrations of drugs for five days before analysis of cell fluorescence in a flow cytometer. Negative and positive controls of proliferation were obtained with actinomycin D-treated or untreated cells, respectively. Each experiment was done twice

To assess the antiproliferative effect of CDK inhibitor R-Roscovitine and its new analogues R-CR8, S-CR8 and MR4, CML cell lines were stained with CFSE and cultured for five days. As shown on Fig. 1, we observed that all tested CDK inhibitors prevent proliferation of all the tested CML cell lines, the Imatinib-sensitive lines as well as the Imatinib-resistant lines. However, and as expected from CDKs inhibition IC_50_ values [20, 21], we observed the three molecules R-CR8, S-CR8 and MR4 act very similarly and display a more than 100-fold stronger antiproliferative effect than Roscovitine (mean IC_50_: 0.22 to 0.24 μM vs 27 μM; see Additional file 1: Table S1).

Moreover, we showed that contrarily to Imatinib, our CDKs inhibitors were always efficient to block the proliferation of metabolic Imatinib-resistant K562-R and KCL22-R cell lines as well as T315I mutation bearing BaF3 cell line whereas Imatinib did not at such low concentrations (Fig. 1, graphs b, d, f).

### CR8 and MR4 block cell cycle mostly in G_2_/M transition

**Fig. 2:**
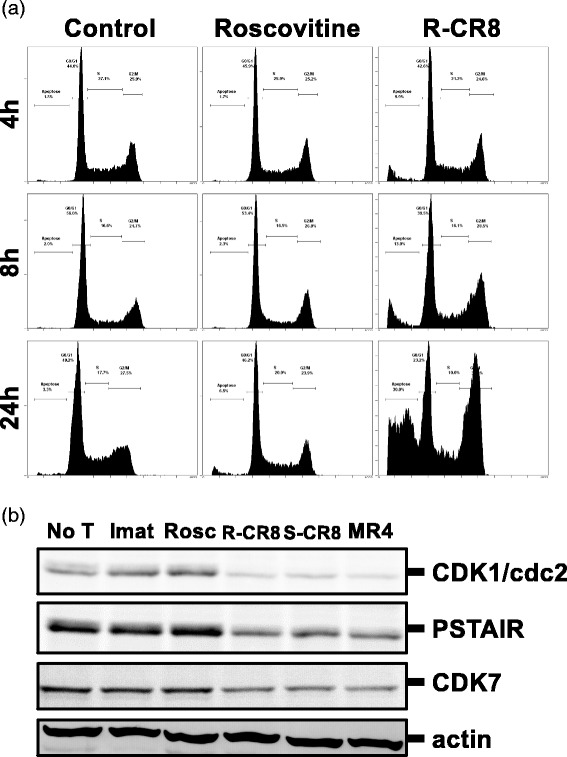
Analysis of cell cycle in KCL22 cell line. **a** Cells were treated or not by 1 μM Roscovitine or R-CR8 for increasing times and distribution of cells among the different phases of cell cycle was assessed by DNA content staining with PI (FL2-log). **b** Western-blotting analysis of CDK1/cdc2, PSTAIR-containing CDKs and CDK7 after 24 h treatment of KCL22 cells. Actin was used as a control of sample loading. *No T* not treated, *Imat* Imatinib, *Rosc* Roscovitine

To investigate the antiproliferative effects of our molecules, we analyzed the cell cycle phase distribution after treatment by CDKs inhibitors. As shown in Fig. 2a for the KCL22 cell line as a representative experiment, R-CR8 blocked cell cycle in G_2_/M transition phase in a time-dependent manner whereas R-Roscovitine was unable. Moreover, G_2_/M blocking of cell cycle was accompanied with an increasing sub-G_0_/G_1_ peak most probably corresponding to apoptotic cells. Similar results were obtained for S-CR8 and MR4 (data not shown). This arrest of cell cycle appeared to be dose- and time-dependent and to begin from 4 h after induction of treatment. Western-blotting analysis of KCL22 treated cells (Fig. 2b) revealed a loss of CDK1/cdc2, PSTAIR motif containing CDKs and CDK7 with CR8 isomers and MR4 that is in accordance with G_2_/M block of the cell cycle and previous studies [23].

### CR8 and MR4 trigger cytotoxic effects in CML cell lines

**Fig. 3:**
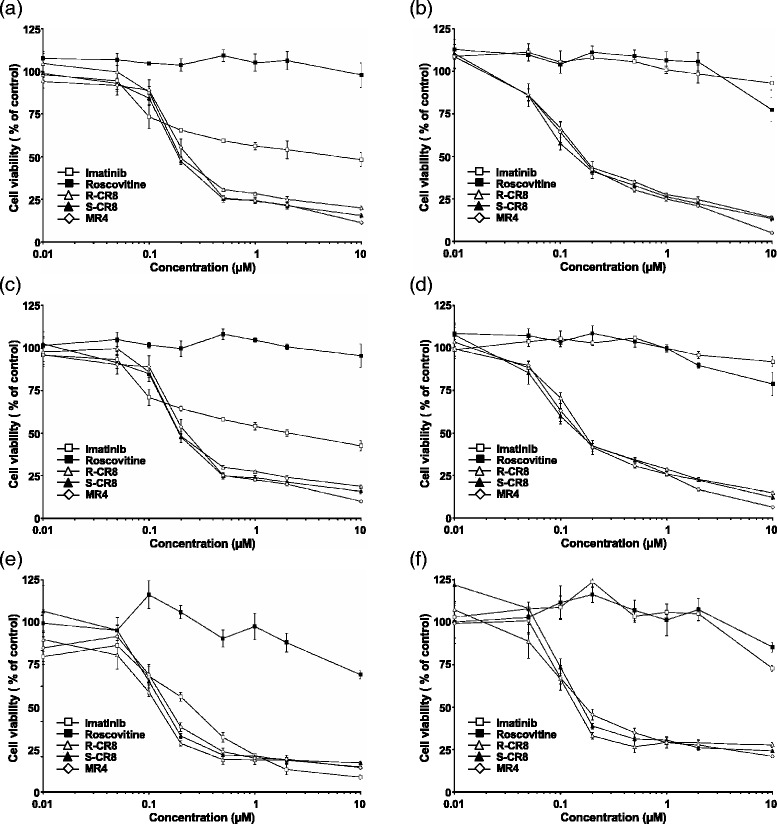
Analysis of cell-viability of Imatinib-sensitive and –resistant CML cell lines. Imatinib-sensitive K562 (**a**), KCL22 (**c**), and BaF3 Bcr-Abl WT (**e**) and Imatinib-resistant K562-R (**b**), KCL22-R (**d**), and BaF3 Bcr-Abl T315I (**f**) CML cell lines were treated or not with increasing concentrations of drugs for two days before measurement of cell metabolism through XTT reduction. Results are expressed as percent of remaining viability *versus* untreated control. Average ± standard deviation of three independent experiments performed in triplicate wells

Based on the appearance of a sub-G_0_/G_1_ peak during cell cycle experiment, we further investigated the cytotoxic effects of CDKs inhibitors on CML cell lines by XTT reduction assay and flow cytometry analysis of double Annexin-V and propidium iodide staining. Imatinib was tested in parallel for comparison. XTT reduction assay, performed 48 h after exposure to increasing concentrations of each compound, showed that all three compounds R-CR8, S-CR8 and MR4 reduced cell survival in a dose-dependent manner on the six CML cell lines tested (Fig. 3). As for antiproliferative effects, R-CR8, S-CR8 and MR4 were roughly equipotent at inducing cell death (mean IC_50_: 1.05, 1.10 and 0.78 μM, respectively) and approximately 30 to 40-fold more potent than R-Roscovitine (mean IC_50_: >36 μM; see Additional file 2: Table S2).

**Fig. 4:**
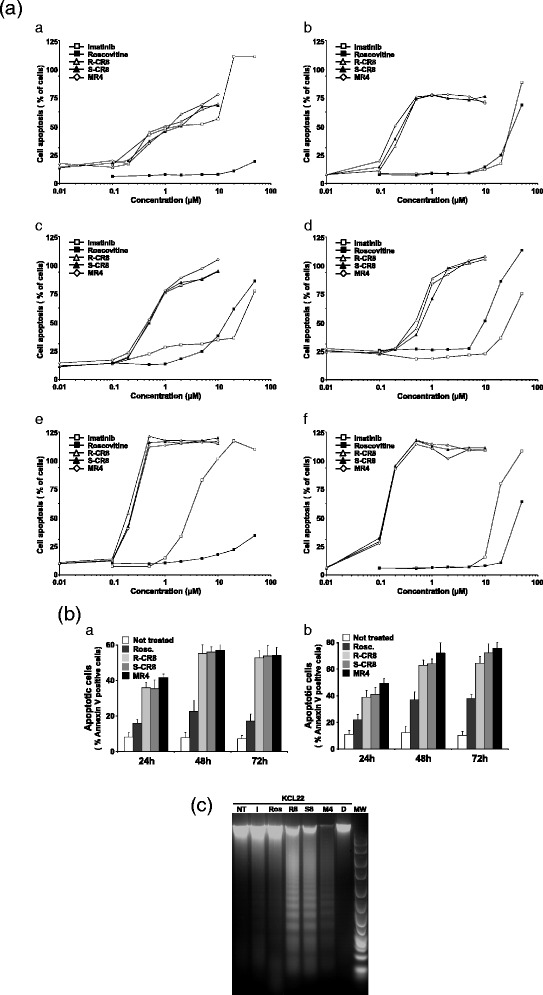
Assessment of apoptosis as cell death mechanism induced by CDKs inhibitors. **a** Analysis of Annexin V-positive cells on Imatinib-sensitive K562 (a), KCL22 (c), and BaF3 Bcr-Abl WT (e) and Imatinib-resistant K562-R (b), KCL22-R (d), and BaF3 Bcr-Abl T315I (f) CML cell lines treating with increasing concentrations of drugs for 48 h. Negative and positive controls of cell viability were obtained with etoposide-treated or untreated cells, respectively. **b** Analysis of K562 and KCL22 Annexin V-positive cells treated for 24, 48 or 72 h with 10 μM drugs. **c** DNA fragmentation analysis of KCL22 cells treated by CDK inhibitors. *NT* not treated, *I* Imatinib, *R* Roscovitine, *R8* R-CR8, *S8* S-CR8, *MR4* MR4, *D* DMSO, *MW* molecular weight

Annexin-V/PI staining assays confirmed the dose-dependent cell death-inducing effects of these CDKs inhibitors with similar IC_50_ values (Fig. 4a). Moreover, additional time-course experiments (Fig. 4b) demonstrated that cytotoxic effects of drugs were time-dependent with maximal response obtained from 48 h exposure time on K562 and KCL22 cells.

Very similar results obtained with XTT reduction assays and Annexin-V/PI staining suggest that the mechanism underlying CR8 isomers- and MR4-induced cell death implied an apoptotic process. Then, we ran a DNA fragmentation analysis on agarose gel electrophoresis. As illustrated in Fig. 4c for the KCL22 cell line, we clearly observed a DNA laddering profile constituted of multiple-180 bp fragments for R-CR8-, S-CR8-, and MR4-treated cells. This profile of internucleosomal fragmentation of genomic DNA is a characteristic hallmark of apoptosis,confirming that CDKs inhibitors-induced cell death is mediated by apoptosis. At the concentrations used, no effect of either Imatinib or R-Roscovitine was observed.

### CR8 and MR4 induce caspase-dependent apoptosis of CML cell lines

**Fig. 5:**
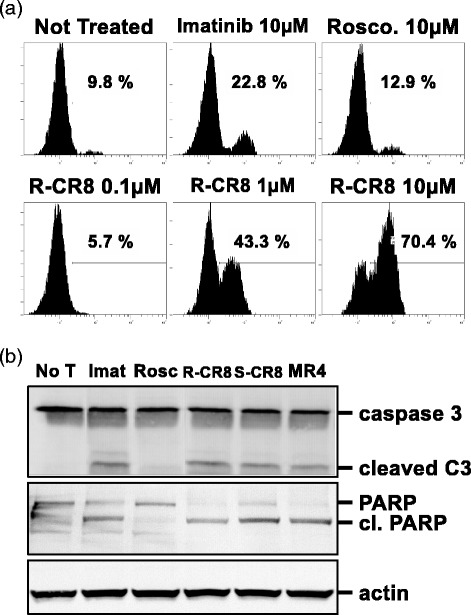
Involvement of caspases processing in CDKs inhibitors-induced apoptosis in KCL22 cells. **a** Cells were left untreated or treated with Imatinib or Roscovitine (10 μM), or R-CR8 (0.1, 1 or 10 μM) before detection of cleaved caspase 3 by flow cytometry. **b** KCL22 cellular extracts were analyzed for caspase 3 and PARP cleavage after 10 μM treatment by drugs. Actin was used as a control of sample loading. *No T* not treated, *Imat* Imatinib, *Rosc* Roscovitine

To determine whether cell apoptosis involved the caspases, we examined processing of late events of apoptosis: cleavage of caspase 3 and PARP by flow cytometry and western-blotting. Results obtained for KCL22 cells are presented in Fig. 5. Cells treated with R-CR8 were stained with anti-cleaved caspase 3 fragment antibody in a dose-dependent manner (from 5.7 % at 0.1 μM to 43.3 % at 1 μM and 70.4 % at 10 μM), whereas cells treated with Roscovitine or untreated were not (Fig. 5a), indicating that R-CR8 specifically induced processing of caspase 3 depending on the concentration and relying it on proapoptotic IC_50_ values. Similar results were obtained for S-CR8 and MR4 (data not shown). Blot analysis of KCL22 cells showed the presence of the cleaved fragments of caspase 3 (15 kDa) and PARP (60 kDa) respectively, only when cells were treated with Imatinib, R-CR8, S-CR8 and MR4 (Fig. 5b). Altogether, these results confirmed that CDK inhibitors induced-apoptosis is caspase-dependent.

**Fig. 6:**
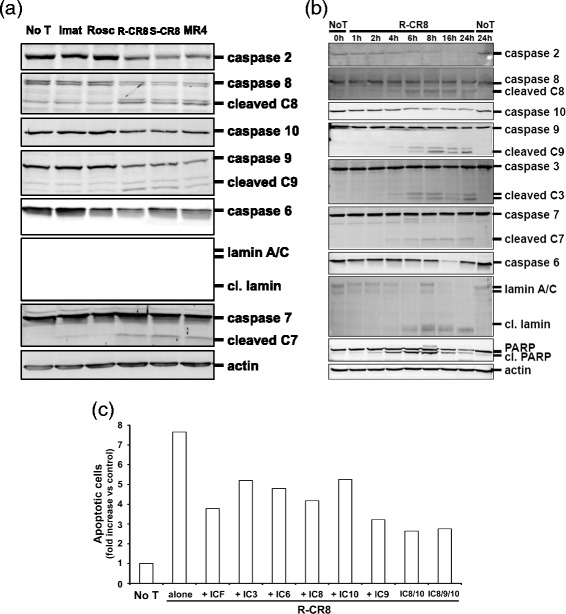
Assessment of extrinsic- and mitochondrial-pathways in apoptosis induced by CDKs inhibitors in KCL22 cells. **a** KCL22 cells were treated with 10 μM of drugs for 24 h before western-blotting analysis of initiator and effector caspases and lamin A/C. **b** Kinetics of apoptotic process induced by Roscovitine-derived CDKs inhibitors. Time-dependent cleavage of caspases revealed by western-blotting analysis of KCL22 cells treated by 10 μM R-CR8 for indicated times. Actin was used as a sample loading control. **c** Analysis of KCL22 Annexin V-positive cells pretreated with 100 μM of caspase inhibitors then treated for 24 h with 10 μM R-CR8. Results are expressed as apoptotic cells fold increase *versus* negative untreated cells. Experiment was done twice

We then tried to determine whether an initiator caspase preceded caspase 3 cleavage. As demonstrated in Fig. 6a, R-CR8 or S-CR8 as well as MR4 treatment of CML cell lines induced activation of initiator procaspases 2, 8, 9 and 10, as well as other effector caspases 6 and 7. These results indicate that both extrinsic and mitochondrial pathways of apoptosis were triggered by the CDKs inhibitors. To investigate if one or some of the initiator procaspases was cleaved first and so, could be considered as the key event in drugs induced apoptosis, we analyzed the kinetics of caspases activation. As illustrated in Fig. 6b for the R-CR8 molecule on KCL22 cells, we observed by western blot experiments that all caspase events were visible from 4 h exposure of cells to the drugs. At this time, all studied caspases showed activation so we were unable to depict the scheme of apoptosis induction by these Roscovitine-derived molecules. Then, we performed Annexin V/PI staining experiments by flow cytometry, using specific caspases inhibitors. Figure 6c shows that pretreating cells with these caspases inhibitors only partially prevented R-CR8 induced apoptosis by less than 50 % for caspases 8 (z-IETD-fmk), 9 (z-LEHD-fmk), and 10 (z-AEVD-fmk) inhibitors and by approximately 70 % when these inhibitors were combined. These results seem to indicate that none of the extrinsic or mitochondrial pathways was predominant for apoptosis induction by the CDKs inhibitors. Moreover, the use of caspase 3 specific (z-DEVD-fmk), caspase 6 specific (z-VEID-fmk) as well as pan-caspase (z-VAD-fmk) inhibitors did not allow to prevent cell death, suggesting that CDKs inhibitors induced-apoptosis acts partially by a caspase-independent pathway.

### CDKs inhibitors provoke loss of mitochondrial membrane potential, down-regulation of Mcl-1, XIAP and survivin and nuclear translocation of AIF

**Fig. 7:**
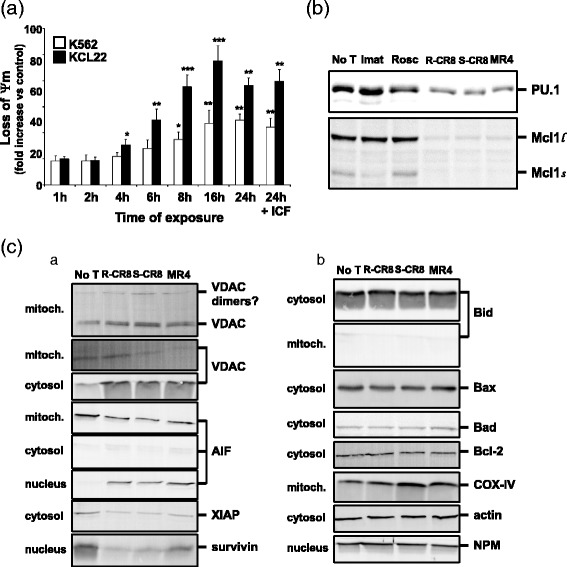
Mitochondrial events implicated in CDKs inhibitors-induced apoptosis. **a** Measurement of mitochondrial membrane potential (MMP, Ψm) variation. K562 or KCL22 cells were treated by 2 μM of the different CDK inhibitors for increasing exposure times and analyzed for MMP variation by DiOC_6_ incorporation in a flow cytometer. Pretreatment was done with 100 μM z-VAD-fmk pan-caspase inhibitor (ICF) when mentioned. Graph shows results for R-CR8, as a representative for other CDK inhibitors. Results are expressed as Ψm fold increase *versus* untreated cells for each time point. Each experiment was performed twice. *: *ρ* < 0.05, **: *ρ* < 0.01 ***: *ρ* < 0.001. **b** Mcl-1 down-regulation assessed by western-blotting on KCL22 cellular extracts. **c** Subcellular fractions of KCL22 cells treated by 10 μM R-CR8, S-CR8, MR4 or left untreated for 24 h were analyzed by western-blotting for pro- and anti-apoptotic molecules. COX-IV, actin, and NPM were used as control for sample loading for mitochondrial, cytosolic, and nuclear fractions, respectively

As demonstrated by DiOC_6_ incorporation on Fig. 7a, the Roscovitine-derived CDKs inhibitors induced reduction of the mitochondrial membrane potential of K562 and KCL22 cell lines in a time-dependent manner. Loss of Ψm appeared significant after 4 h exposure to drugs and increased up to five-fold the control level after 16 h exposure on KCL22 cell line which seemed to be more sensitive than K562, in accordance with previous results on cytotoxic activities. Pretreatment of cells with the pan-caspase inhibitor z-VAD-fmk was absolutely unable to reverse loss of mitochondrial membrane potential, indicating this event precedes subsequent caspase cascade.

Then, we evaluated the expression of Mcl-1 and its transcription factor PU.1 at the protein level. Western blot revealed a pronounced decrease in both Mcl-1 and PU.1 proteins when cells were treated by CDKs inhibitors (Fig. 7b), removing the anti-apoptotic function of Mcl-1 on the mitochondria. Conjugated to this, subcellular fractionation analysis revealed the release of cytochrome c from mitochondria to cytosol where it could complex with Apaf-1 and caspase 9 to form the apoptosome. This release of cytochrome c (and other apoptogenic factors) from the mitochondria act through the Voltage Dependent Anion Channel (VDAC) whose dimers were possibly visualized (Fig. **7**c).

Translocation of AIF from mitochondria to nucleus (although not seen in the cytosol), where it could induce DNA fragmentation as early evidenced, also occurred consequently to Ψm loss. This observation reinforced the idea of a caspase-dependent and –independent contribution to apoptosis triggered by R-CR8, S-CR8 and MR4 treatment of CML cell lines, and thus would explain the relatively modest effects of caspases inhibitors observed in our experiments.

Additionally, we were unable to detect the truncated form of Bid in cellular extracts (neither cytosol nor mitochondria), suggesting that extrinsic and mitochondrial pathways of apoptosis are possibly acting independently.

Moreover, XIAP and survivin, two survival and antiapoptotic molecules belonging to the Inhibitor of Apoptosis Proteins (IAPs) family, were both strongly down-regulated from their subcellular location, cytosol and nucleus, respectively. In contrast, no changes were observed for other pro- or anti-apoptotic molecules, such as Bax, Bad, and Bcl2 (Fig. 7c).

### CR8 and MR4 induce ROS generation consequently to caspases activation

**Fig. 8:**
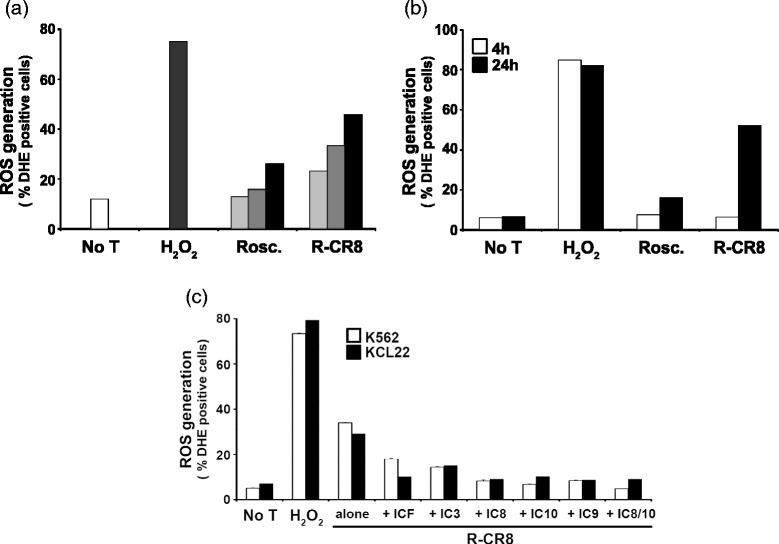
Effects of CDKs inhibitors on ROS generation in CML cell lines. **a** ROS were detected by DHE probe on KCL22 cells treated with increasing concentrations of Roscovitine or R-CR8: 1 μM (*light grey*), 2 μM (*dark grey*), and 10 μM (*black*). B, KCL22 were treated by 10 μM Roscovitine or R-CR8 for 4 h (*white bars*) or 24 h (black bars) before measurement of ROS. C, K562 (*white bars*) and KCL22 (*black bars*) cells were treated by 2 μM R-CR8 after pretreatment with 50 μM caspase inhibitors. All results are expressed as percent of DHE-positive cells

We next examined if cell treatment with the CDKs inhibitors induced the generation of reactive oxygen species within the mitochondria. As shown in Fig. 8, R-CR8 induced the production of ROS in a concentration- and time-dependent manner in the KCL22 cell line. ROS were undetectable within the 4 h while apoptosis already occurred. Then, we measured ROS generation on cells pretreated by caspases inhibitors. Figure 8c shows that ROS were abundantly prevented with initiator caspase inhibitors and to a lesser extent with caspase 3 and pan-caspase inhibitors, suggesting that ROS generation simply represent a secondary event of the apoptotic process.

## Discussion

Development of drug resistance, leading to selection of resistant cell clone, is often a major obstacle for successful cancer treatment. Therefore, there is always an urgent need for novel molecules with improved efficacy against tumor cells, even in currently “curable” diseases. Previous reports indicated that CDKs not only regulate eukaryotic cellular proliferation, but also participate in multiple cellular processes such as transcription [24]. Therefore, inhibition of CDKs offers a promising therapeutic strategy against cancer [25].

The aim of the present study was to analyze whether analogues of Roscovitine, a well-known CDKs inhibitor, could affect cell death and survival of CML cell lines and to decipher the mechanisms of action of these molecules. We have shown that the R-CR8, S-CR8 and MR4 analogues exert growth-inhibitory effects in all CML cell lines, both Imatinib-sensitive and Imatinib-resistant, by increase of the G_2_/M phase of the cell cycle (Figs. 1 and 2). This G_2_/M blockade is sustained by a down-regulation of CDK1 and CDK2 as previously demonstrated for Roscovitine, depending on cell types [14, 26]. Observed inhibition of CDK7 would also be indirectly implicated since this kinase phosphorylates threonine residues on and then activates CDK1 and CDK2 [27, 28].

In addition to their cytostatic properties, Roscovitine analogues have been shown to trigger apoptosis on CML cell lines with IC_50_ values in the micromolar range. Although analogues displayed only approximately 2 to 4-fold better affinity for CDKs [20], antiproliferative and pro-apoptotic effects of these molecules were 100- and 30-fold more potent than Roscovitine, respectively. Such differences could be explained by slight differences in cell permeability to drugs, intracellular stability, or distribution across cell organelles. Another possibility is that Roscovitine analogues bind to other yet unidentified targets (maybe a kinase or not) with a 10- to 100-fold stronger affinity than Roscovitine does.

To delineate mechanisms of the cytotoxic effects of R-CR8, S-CR8 and MR4, we have tested whether observed CML cell lines apoptosis involved caspase activation and, if so, which pathway (the extrinsic- or mitochondrial one) was predominant for this activity. In this study, treatment of CML cells with the Roscovitine-derived inhibitors leads to increase of active forms of caspase 3 and its substrate PARP ultimately leading to cell death. Kinetics analysis of caspase activation (Fig. 6b) revealed that processing of the initiator as well as of the effector caspases occurred at the same time, from 4 h of treatment. This observation was consistent with kinetics previously reported for leukemia cells [29]. Nevertheless, it is surprisingly that all initiator and effector caspases were activated simultaneously, and thus, it does not permit to identify the former caspase event. Experiments using specific caspase inhibitors brought us some new information. First, we found that the pan-caspase inhibitor, as well as specific caspase inhibitors, used at a high concentration (100 μM), were only partially protective against cell death processing (Fig. 6c). In contrast, some previous studies on CML cell lines using Cepharantine demonstrated classic caspase-dependent apoptotic responses and were easily blocked by only 20 μM Z-VAD-fmk [30]. This limited protection suggests that R-CR8, S-CR8 and MR4 also trigger caspase-independent cell death. Second, ΔΨm loss, also significantly appearing from 4 h treatment, was completely unchanged under pan-caspase inhibitor pretreatment (Fig. 7a). This led us to assume that caspases are activated downstream of mitochondria events. Taken together, these results support that the hypothesis that CR8 isomers- and MR4-mediated apoptosis begins with the commitment of mitochondrial events and that cell death proceeds through caspase-dependent and -independent pathways, suggesting that caspase cascade may be important to amplify the apoptotic signal emanating from mitochondria, but not absolutely crucial to achieve cell death, as previously reported [31, 32].

Bid is a substrate of caspase-8 in the extrinsic pathway, and provides a link between the extrinsic and mitochondrial pathways of apoptosis. We then considered the possibility that caspase 8 activation could be responsible for amplifying caspase 9 activation through cleavage of Bid [33], although this seemed unlikely given that activation of caspase 9 appeared to occur concomitantly with caspase 8 activation. Indeed, CDKs inhibitors treatment was not associated with detectable Bid cleavage (Fig. **7**c). This suggests that extrinsic- and mitochondria-pathways of caspase activation are not linked in this model. However, we cannot rule out a technical problem in the detection of truncated Bid.

According to current knowledge, and consistent with above-mentioned results, cytotoxic drugs cause apoptosis mainly through the mitochondrial pathway. In this pathway, apoptosis is induced by an intrinsically generated death signal which arrives at mitochondria, causing loss of Ψm and release of cytochrome c and/or AIF into the cytoplasm [34, 35].

The possibility that Roscovitine analogues act directly on mitochondria permeability transition was discarded by previous studies on isolated mitochondria [36]. A factor leading to cytochrome c release consequently to ΔΨm loss and VDAC opening is the antiapoptotic factor Mcl-1, located on the outer membrane of mitochondria. Mcl-1 plays a critical role in negatively modulating mitochondrial apoptotic events, such as cytochrome c release and caspase activation [37]. Our results show that Mcl-1 and its transcription factor PU.1 were drastically down-regulated under R-CR8, S-CR8, and MR4 treatment, confirming works on Mcl-1 in other malignancies such as chronic lymphocytic leukemia [38], neuroblastoma [39], or multiple myeloma [40]. Previous reports have shown that RNA polymerase II phosphorylation by CDK7 and CDK9 is sensitive to Roscovitine or R-CR8 treatment and that its inhibition leads to suppressed transcription [17, 20]. So far, observed down-regulation of Mcl-1 certainly results from this CDK7/CDK9-mediated inhibition of transcription and acts as the leading event to subsequent release of cytochrome c. Moreover, we also observed a ~64 kDa protein band revealed by anti-VDAC antibody, possibly corresponding to VDAC homodimers that assemble between Mcl-1 decrease and cytochrome c release [41, 42].

It has recently been shown that Mcl-1 is required for survival during BCR-ABL transformation and in established BCR-ABL(+) leukaemia [43]. Thus, down-regulation of Mcl-1 triggered by Roscovitine analogues would suppress the survival advantage conferred by Mcl-1 in chronic myeloid leukemia, resulting in apoptotic cell death.

Among other cellular proteins regulating caspase activation are the IAPs (Inhibitor of Apoptosis Proteins), including XIAP and survivin [44]. Similarly to Mcl-1, we found that both survivin and XIAP were strongly down-regulated after R-CR8, S-CR8 and MR4 treatment (Fig. 7). Survivin acts as a prosurvival and antiapoptotic factor by inhibiting active forms of caspase-3 and −7 and Bax- and Fas-induced apoptotic pathways [45], and by activating mitosis and cytokinesis during the G_2_/M phase [46]. Thus, down-regulation of survivin may be triggered by CDK1/CDK2 impairment during cell cycle as well as CDK7/CDK9-mediated transcription inhibition. XIAP directly neutralizes initiator caspase 9 and effector caspases 3 and 7 through its baculovirus-IAP-repeat domains 3 and 2, respectively [47]. Study of survivin and XIAP genes in CML revealed that disease progression from chronic to blastic phases was accompanied with overexpression of survivin [48] and XIAP mRNA levels [49, 50]. Considering this, survivin and XIAP would represent another targets to control disease progression, arguing in favour of the evaluation of CDKs inhibitors in CML.

As discussed above, our results using synthetic caspases inhibitors strongly suggest that CDKs inhibitors-mediated cell death of CML cell lines also occurs through caspase-independent pathway. Such pathway is described to proceed from mitochondria release of non caspase-dependent factors such as AIF or Endonuclease G [36, 51]. Subcellular fractionation demonstrated the translocation of propapoptotic factor AIF from mitochondria to nucleus (despite the lack in the cytosol) where it could induce DNA fragmentation (Figs. 4 and 7), confirming that Roscovitine analogues-induced cell death implies both caspase-dependent and -independent pathways.

Finally, we quickly assessed the Roscovitine analogues ability to induce ROS generation. We showed that ROS production was induced by CDKs inhibitors in a time- and dose-dependent manner. However, ROS generation was considerably delayed behind other events of apoptosis induction (Figs. 6 and 8). This suggests that ROS generation by CDKs inhibitors was only a consequence of cell death processing. Surprisingly abolition of ROS was observed with caspases inhibitors, suggesting that ROS generation could only be a hallmark of caspase-dependent CDKs inhibitors-mediated apoptosis. These results contrast with most reported studies where ROS generation seems to precede and to trigger mitochondrial commitment. However, there is still debate for place and role of free radical production in the apoptotic process, which may vary with stimulus and cell type. In some studies, induction of apoptosis by chemotherapeutic agents has been linked to production of ROS [52, 53], whereas other studies have suggested that ROS generation represents a consequence rather than a cause of cell death [54]. Moreover, results obtained here are contradictory with one of two recent reports indicating that Roscovitine treatment of c-Abl-activated neutrophils in inflammatory context, drive them to apoptosis with a markedly decrease in ROS generation [55]. However, in the breast cancer context, Roscovitine induces apoptosis by increasing ROS [56]. Further works, in particular using ROS scavenger, will be needed to delineate ROS contribution to Roscovitine analogues effects in CML context.

## Conclusions

The data presented here demonstrate that prolonged exposure of human CML cell lines to novel CDKs inhibitors R-CR8, S-CR8, and MR4 represents a potent stimulus for mitochondrial damage and apoptotic cell death of these cells, targeting and/or inducing down-regulation of key molecules sustaining disease establishment and evolution. These second-generation analogues of Roscovitine should now be investigated for their toxicity and antitumor properties in appropriate animal models of CML. Remaining questions about cell death mechanisms should also be investigated.

## Additional files

Additional file 1: Table S1. IC_50_ (μM) antiproliferative effect on Imatinib-sensitive or –resistant cell lines. Description: this table summarizes IC_50_ of antiproliferative effect of CDK inhibitors obtained on all tested CML cell lines. Additional file 2: Table S2. IC_50_ (μM) propapoptotic effect on Imatinib-sensitive or –resistant cell lines. Description: this table summarizes IC_50_ of proapoptotic effect of CDK inhibitors obtained on all tested CML cell lines.
