# Supplementary material for: Antitumoral effects of cyclin-dependent kinases inhibitors CR8 and MR4 on chronic myeloid leukemia cell lines
Source: J Biomed Sci. 2015 Jul 17;22(1):57. doi: 10.1186/s12929-015-0163-x (PMC4504225; doi:10.1186/s12929-015-0163-x)
Supplement: Additional file 1: Table S1. — IC50 (μM) antiproliferative effect on Imatinib-sensitive or –resistant cell lines. Description: this table summarizes IC50 of antiproliferative effect of CDK inhibitors obtained on all tested CML cell lines. [file 12929_2015_163_MOESM1_ESM.doc]

| **Supplementary Table 1.** IC_50_ (µM) antiproliferative effect on Imatinib-sensitive or –resistant cell lines | | | | | | | | |
| --- | --- | --- | --- | --- | --- | --- | --- | --- |
|  | K562 | K562-R | KCL22 | KCL22-R | BaF3 WT | BaF3 T315I | **Mean** | **Fold increase*** |
| Imatinib | 0.20 | 15 | 0.15 | 8 | 4.50 | 15 | - | - |
| Roscovitine | 35 | 35 | 4 | 12 | 45 | 32 | **27** | **1** |
| R-CR8 | 0.30 | 0.32 | 0.05 | 0.23 | 0.15 | 0.25 | **0.22** | **123** |
| S-CR8 | 0.35 | 0.37 | 0.05 | 0.25 | 0.25 | 0.15 | **0.24** | **113** |
| MR4 | 0.35 | 0.38 | 0.06 | 0.25 | 0.25 | 0.25 | **0.26** | **104** |
| * fold increase is calculated based on Roscovitine IC_50_ set at 100% | | | | | | | | |
