# Supplementary material for: Antitumoral effects of cyclin-dependent kinases inhibitors CR8 and MR4 on chronic myeloid leukemia cell lines
Source: J Biomed Sci. 2015 Jul 17;22(1):57. doi: 10.1186/s12929-015-0163-x (PMC4504225; doi:10.1186/s12929-015-0163-x)
Supplement: Additional file 2: Table S2. — IC50 (μM) propapoptotic effect on Imatinib-sensitive or –resistant cell lines. Description: this table summarizes IC50 of proapoptotic effect of CDK inhibitors obtained on all tested CML cell lines. [file 12929_2015_163_MOESM2_ESM.doc]

| **Supplementary Table 2.** IC_50_ (µM) proapoptotic effect on Imatinib-sensitive or –resistant cell lines | | | | | | | | |
| --- | --- | --- | --- | --- | --- | --- | --- | --- |
|  | K562 | K562-R | KCL22 | KCL22-R | BaF3 WT | BaF3 T315I | **Mean** | **Fold increase*** |
| Imatinib | 11 | 15 | 12 | 35 | 3 | 15 | - | - |
| Roscovitine | >50 | 12 | 45 | 12 | >50 | 45 | **>36** | **1** |
| R-CR8 | 4 | 0.60 | 0.40 | 0.60 | 0.20 | 0.25 | **1.01** | **>36** |
| S-CR8 | 4 | 0.70 | 0.40 | 0.80 | 0.25 | 0.15 | **1.05** | **>34** |
| MR4 | 2.5 | 0.80 | 0.30 | 0.60 | 0.25 | 0.25 | **0.78** | **>46** |
| * fold increase is calculated based on Roscovitine IC_50_ set at 100% | | | | | | | | |
